# An *in silico—in vitro* Pipeline Identifying an HLA-A^*^02:01^+^ KRAS G12V^+^ Spliced Epitope Candidate for a Broad Tumor-Immune Response in Cancer Patients

**DOI:** 10.3389/fimmu.2019.02572

**Published:** 2019-11-15

**Authors:** Michele Mishto, Artem Mansurkhodzhaev, Ge Ying, Aruna Bitra, Robert A. Cordfunke, Sarah Henze, Debdas Paul, John Sidney, Henning Urlaub, Jacques Neefjes, Alessandro Sette, Dirk M. Zajonc, Juliane Liepe

**Affiliations:** ^1^Centre for Inflammation Biology and Cancer Immunology (CIBCI) & Peter Gorer Department of Immunobiology, King's College London, London, United Kingdom; ^2^Charité – Universitätsmedizin Berlin, Corporate Member of Freie Universität Berlin, Humboldt-Universität zu Berlin, Berlin Institute of Health, Institut für Biochemie, Berlin, Germany; ^3^Quantitative and Systems Biology, Max-Planck-Institute for Biophysical Chemistry, Göttingen, Germany; ^4^Division of Immune Regulation, La Jolla Institute for Allergy and Immunology, La Jolla, CA, United States; ^5^Department of Immunohematology and Bloodbank, Leiden University Medical Center LUMC, Leiden, Netherlands; ^6^Division of Vaccine Discovery, La Jolla Institute for Allergy and Immunology, La Jolla, CA, United States; ^7^Bioanalytical Mass Spectrometry Group, Max-Planck-Institute for Biophysical Chemistry, Goettingen, Germany; ^8^Institut for Clinical Chemistry, University Medical Center Goettingen Bioanalytics, Goettingen, Germany; ^9^Department of Cell and Chemical Biology, Oncode Institute, Leiden University Medical Center LUMC, Leiden, Netherlands; ^10^Department of Medicine, University of California, San Diego, San Diego, CA, United States; ^11^Division of Immune Regulation, La Jolla Institute for Immunology, La Jolla, CA, United States; ^12^Department of Internal Medicine, Faculty of Medicine and Health Sciences, Ghent University, Ghent, Belgium; ^13^Department of Life Sciences, Centre for Integrative Systems Biology and Bioinformatics, Imperial College London, London, United Kingdom

**Keywords:** proteasome, peptide splicing, adoptive T cell therapy targets, antigen presentation, cancer epitopes, KRAS, tumor immunology

## Abstract

Targeting CD8^+^ T cells to recurrent tumor-specific mutations can profoundly contribute to cancer treatment. Some of these mutations are potential tumor antigens although they can be displayed by non-spliced epitopes only in a few patients, because of the low affinity of the mutated non-spliced peptides for the predominant HLA class I alleles. Here, we describe a pipeline that uses the large sequence variety of proteasome-generated spliced peptides and identifies spliced epitope candidates, which carry the mutations and bind the predominant HLA-I alleles with high affinity. They could be used in adoptive T cell therapy and other anti-cancer immunotherapies for large cohorts of cancer patients. As a proof of principle, the application of this pipeline led to the identification of a KRAS G12V mutation-carrying spliced epitope candidate, which is produced by proteasomes, transported by TAPs and efficiently presented by the most prevalent HLA class I molecules, HLA-A^*^02:01 complexes.

## Introduction

Activating CD8^+^ T cells to recurrent tumor-specific mutations is one of a number of cutting-edge strategies to treat cancer. It can be achieved by immunotherapy approaches such as adoptive T cell therapy (ATT), peptide vaccination and dendritic cell (DC) vaccination. Neoepitopes that carry cancer recurrent mutations and efficiently bind common Human Leukocyte Antigen class I (HLA-I) variants are ideal targets for tumor immunology vaccination of large cohorts of patients.

Peptide epitopes are generally produced by proteasomes, which are the final effectors of the ubiquitin-proteasome system (1). Epitope production is the first step of the antigen processing and presentation (APP) pathway, which accounts for the epitope translocating into the endoplasmic reticulum (ER) lumen through mediation by transporters associated with antigen processing (TAPs), binding to the peptide loading complex, trimming by exopeptidases, binding to the HLA-I complex, and transport to the cell surface for recognition by cytotoxic T lymphocytes (CTLs) (2).

There are several proteasome isoforms that can be involved in APP. The most active proteasome isoform is a large (26S) protease consisting of a 20S proteasome core coupled to one or two 19S regulatory complexes. The 19S subunit contains the ubiquitin recognition and removal system as well as an unfolding activity, the 20S form is the actual protease. The 20S proteasome is constituted of four rings; two α rings at the apexes; and two β rings forming the central chamber. Each ring has seven distinct subunits. Each β ring carries three catalytic (i.e., β1, β2, and β5) subunits, which have distinct preferences for peptide sequence motifs (1). Human cells can express different isoforms of catalytic subunits, which are incorporated in distinct proteasome isoforms. Standard proteasomes (s-proteasomes) contain β1, β2, and β5 subunits. Immunoproteasomes (i-proteasomes) contain β1i, β2i, and β5i subunits and are constitutively present in immune cells, such as mature DCs, as well as in cells exposed to an inflammatory milieu (3). Tumors express various intermediate-type proteasome isoforms, in which standard- and immuno-subunits are assembled in one proteasome complex (4, 5). 20S proteasome is also functional alone in cells or coupled to other regulatory subunits such as PA28 αβ (3, 6, 7).

Proteasomes can break proteins and release the peptide fragments or re-ligate them, thereby forming “spliced peptides,” which have sequences that do not recapitulate the linear sequence of the parental protein (3, 8, 9). Spliced peptides may represent a sizeable portion of the peptide pool bound to HLA-I molecules—i.e., the HLA-I immunopeptidome—of tumor and non-tumor human cell lines (10–12). This hypothesis is currently a matter of debate since different analytical approaches obtained discordant results (10–16). According to our previous analysis, however, several antigens displayed by HLA-I immunopeptidomes are represented only by spliced peptides. The antigens represented by spliced peptides seem to be longer, more hydrophobic and more basic than those represented by non-spliced peptides. Antigens that are represented by both spliced and non-spliced peptides show antigenic hot spots, i.e., antigenic areas from which both spliced and non-spliced peptides derive (12). HLA-I-bound spliced peptides are generally less abundant than non-spliced peptides (10–12, 17). Proteasome-generated spliced epitopes can trigger specific CTL responses *ex vivo*/*in vivo* against tumor- and type 1 diabetes-associated antigens (17, 18) as well as pathogens (19). ATT targeting a spliced epitope successfully treated a melanoma patient (20, 21).

**Figure 1:**
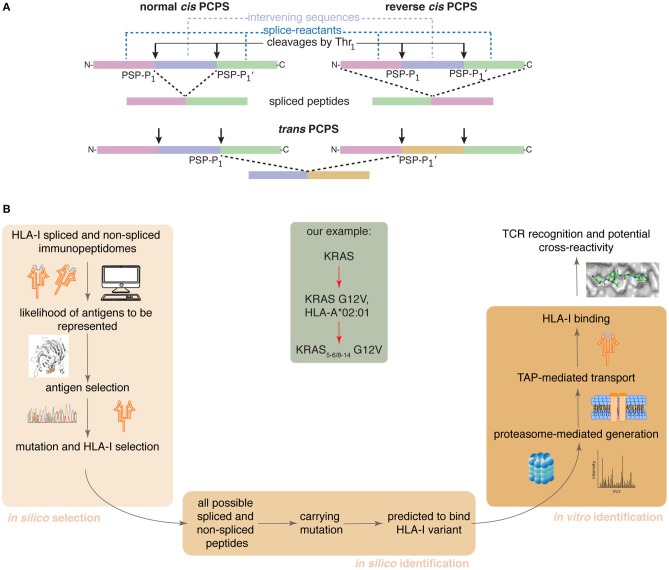
Proteasome-catalyzed peptide splicing and the *in silico/in vitro* pipeline to identify tumor-specific spliced epitope candidates. **(A)** Proteasome-generated spliced peptides can be formed by: (i) *cis* PCPS, when the two splice-reactants derive from the same polypeptide molecule, the ligation can occur in normal order, i.e., following the orientation from N- to C-terminus of the parental protein (normal *cis* PCPS), or in reverse order (reverse *cis* PCPS); (ii) *trans* PCPS, when the two splice-reactants originate from two distinct protein molecules or two distinct proteins. PSP-P1 and PSP-P1' are the C-terminal residue of the N-terminal splice-reactant and the N-terminal residue of the C-terminal splice-reactant, respectively. The splice-reactants are separated by the intervening sequence. **(B)** Representation of the steps of the *in silico* – *in vitro* pipeline proposed here. Through its application, we identified an HLA-A*02:01^+^ KRAS G12V^+^ spliced epitope candidate (center gray frame).

Proteasome-catalyzed peptide splicing (PCPS; see Figure 1A) can occur by combining two non-contiguous sequences of the same molecule (*cis* PCPS) or of two distinct molecules (*trans* PCPS). The latter seems to be efficiently catalyzed *in vitro* by purified proteasomes (22–24) and may constitute a large portion of the HLA-I associated spliced immunopeptidomes (11).

Although the role of spliced peptides in central tolerance still has to be investigated, the theoretically large sequence variability of spliced peptides makes them attractive for anti-cancer immunotherapy (25). Indeed, some of the most recurrent driver mutations in tumors often cannot be efficiently represented by canonical non-spliced peptides bound to the predominant HLA-I variants because of antigen sequence restrictions. On the contrary, they might be represented on the cell surface by tumor-specific spliced epitopes. Therefore, the identification of tumor antigen-specific spliced epitopes might represent a unique opportunity to treat a large cohort of patients.

We here present a pipeline combining *in silico* and *in vitro* approaches. It successfully identifies tumor-specific spliced and non-spliced epitope candidates, which can be further validated as targets for anti-cancer immunotherapies, as illustrated by the HLA-A^*^02:01^+^ KRAS G12V^+^ spliced epitope candidate here described (Figure 1B).

## Materials and Methods

### Antigen Selection and Spliced Epitope Candidate Identification

To rank antigens that are over-represented in HLA-I immunopeptidomes by spliced and non-spliced peptides according to their protein characteristics, we generated a simple model based on the following characteristics: length, hydrophobicity index, isoelectric point, and instability index.

We first calculated these characteristics for all proteins in the Uniprot Reference human proteome database. Next, we analyzed the previously published HLA-I immunopeptidomes of GR-LCL, HCC1143 and HCT116 cell lines (12). All identified spliced and non-spliced peptides were mapped to their antigen(s) of origin, thereby determining a set of represented antigens. Among those antigens not represented in the MS-detected HLA-I immunopeptidomes, there are likely many antigens that would be represented if we considered larger HLA-I immunopeptidome datasets. For this reason, we compared the characteristics of the represented antigens to the characteristics of all proteins (control set). The aim was to determine a combination of the four selected protein characteristics that has the largest difference between the represented antigen set and the control set.

We define the feature sum (*f* ) as: *f* = ∑*p*_*i*_ *c*_*i*_, where *c* is the vector of the four selected features (length, hydrophobicity index, isoelectric point and instability index) and *p* is the vector of factors ranging from −1 to 1. A factor of −1 would favor presentation, while a factor of 1 would disfavor presentation. We use Bayesian inference in a Markov Chain Monte Carlo scheme to determine the factors that result in a distribution of *f* for represented antigens (*F*_1_), which is most different to the distribution of *f* for control proteins (*F*_0_). The difference between the densities *F*_*diff*_ = *F*_1_*-F*_0_ indicates which *f* is favoring (positive values) or disfavoring (negative values) protein representation. Next, we calculated *F*_*diff*_*(f)* for a set of candidate antigens (BRAF, KRAS, HRAS, NRAS, TP53, CDK4, IDH1, TYR) using the Kolmogorov-Smirnov distance. To do so, we sampled (*N* = 1,000) from the posterior distribution of factors resulting in distributions of *F*_*diff*_ for each candidate antigen. Antigens with the highest *F*_*diff*_ have characteristics that lead to more likely representation of those antigens in HLA-I immunopeptidomes.

### Peptide Synthesis and Proteasome Purification

All peptides were synthesized using Fmoc solid phase chemistry (Supplementary Table 2). 20S proteasome was purified from peripheral blood as follows: (i) 10 ml peripheral blood was homogenized, lysed and centrifuged; (ii) the supernatant was fractionated by ammonium sulfate precipitation (35% and then 75%); (iii) the latter pellet was fractioned by chromatography on DEAE-Sephacel; (iv) the selected fractions were separated by 10–40% sucrose gradient and followed by (v) anion exchange chromatography on Mono Q in an Akta-FPLC system; (vi) the selected fractions (2–4 mL) were further purified by DEAE-Affi-gel-blue chromatography. In each of the (ii–vi) steps, the fractions were monitored by degradation assays of standard short fluorogenic substrate Suc-LLVY-AMC. Proteasome concentration was measured by Bradford staining and verified by Coomassie staining of an SDS-Page gel, as shown elsewhere (26). The purity of the preparation using this protocol has been previously shown (27).

### *In vitro* Digestions and MS Measurements

Synthetic polypeptides (40 μM) were digested by 3 μg 20S proteasomes in 100 μl TEAD buffer for different time points (0–20 h) at 37°C, as previously described (27). We performed three independent experiments, each of them measured either 3 times (for the 0–4 h kinetics) or 2 times (for 20 h digestions) by mass spectrometry (MS).

The identification of target peptide products was carried out by targeted MS using a mass to charge ratio (m/z) inclusion list. The inclusion list was comprised of all theoretically possible 8–12 mer spliced and non-spliced peptide products derived from KRAS_2−35_ G12V synthetic polypeptide substrate, which carried the G12V mutation and were predicted to bind HLA-A^*^02:01 complex with IC50 ≤ 100 nM (see below). The same principle was applied for the peptide products derived from the wild type KRAS_2−35_ G12 synthetic polypeptide substrate (Supplementary Table 1). To this end, 20 h *in vitro* digestions with 20S proteasomes were measured by Fusion Lumos Mass Spectrometer (Thermo Fisher Scientific). Prior to measurement, the samples were diluted with the loading buffer (2% acetonitrile, 0.05% Trifluoroacetic acid) containing human insulin (Sigma-Aldrich) to a final substrate concentration of 25 μM and insulin concentration of 2 μM. Insulin was used as a coating polymer to prevent binding of peptides to the glass vials used for measurements and to improve reproducibility between technical replicates. Eight μl of those dilutions (corresponding to 200 pmol of substrate initially present in the sample) were injected. Samples were loaded and separated by a nanoflow HPLC (RSLC Ultimate 3000) on an Easy-spray C18 nano column (30 cm length, 75 μm internal diameter) coupled on-line to a nano-electrospray ionization Fusion Lumos mass spectrometer (Thermo Fisher Scientific). Peptides were eluted with a linear gradient of 5–55% buffer B (80% ACN, 0.1% formic acid) over 88 min at 50°C at a flow rate of 300 nl/min. The instrument was programmed within Xcalibur 3.1.66.10 to acquire MS data in a Data Dependent Acquisition mode using Top 20 precursor ions. We acquired one full-scan MS spectrum at a resolution of 120,000 with an automatic gain control (AGC) target value of 1,000,000 ions and a scan range of 300–1,600 m/z with maximum injection time set to 50 ms and intensity threshold set to 50,000. The MS/MS fragmentation was conducted using HCD collision energy (35%) with an orbitrap resolution of 30,000 at 1.4 m/z isolation window with Fixed First Mass set to 105 m/z. The AGC target value was set up at 100,000 with a maximum injection time of 128 ms. A dynamic exclusion of 30 s and 1–7 included charged states were defined within this method.

*In vitro* proteasome-mediated digestion kinetics (0–4 h) and the 20 h digestions were measured by LC-MS/MS as follows: Prior to measurement, samples were diluted with the loading buffer and insulin as described above. Eight μl (i.e., 200 pmol substrate) of those dilutions were loaded. Samples were loaded and separated by a nanoflow HPLC (RSLC Ultimate 3000) on an Easy-spray C18 nano column (30 cm length, 75 μm internal diameter; Dr. Maisch) coupled on-line to a nano-electrospray ionization Q Exactive Hybrid-Quadrupol-Orbitrap mass spectrometer (Thermo Fisher Scientific). Peptides were eluted with a linear gradient of 5–55% buffer B (80% ACN, 0.1% formic acid) over 88 min at 50°C at a flow rate of 300 nl/min. The instrument was programmed within Xcalibur 3.1.66.10 to acquire MS data in a Data Dependent Acquisition mode using Top 20 precursor ions. We acquired one full-scan MS spectrum at a resolution of 70,000 with an automatic gain control (AGC) target value of 1,000,000 ions and a scan range of 350~1,600 m/z. The MS/MS fragmentation was conducted using HCD collision energy (30%) with an Orbitrap resolution of 35,000 at 2 m/z isolation window with Fixed First Mass set to 110 m/z. The AGC target value was set up at 100,000 with a maximum injection time of 128 ms. For Data Dependent Scans the minimum AGC target value and the Intensity threshold were set to 2,600–20,000 accordingly. A dynamic exclusion of 25 s and 1–6 included charged states were defined within this method.

### Spliced and Non-spliced Peptide Identification and Quantification As Well as Computation of SCS-P1 and PSP-P1

Peptides were identified using the Mascot version 2.6.1 (Matrix Science) search engine. Mass spectra were searched against a customized database that includes all theoretically possible spliced and non-spliced peptides (28). M oxidation, N-terminal acetylation and NQ deamidation were set as variable modification. For the peptide identification in the Orbitrap Q Exactive measurements, we set as mass tolerances for MS and MS/MS 6 ppm and 20 ppm, respectively. For the peptide identification in the Fusion Lumos measurements, we set as mass tolerances for MS and MS/MS 5 ppm and 0.03 Da, respectively.

Peptide hits were filtered using an ion score cut-off of 20, a *q*-value cut off of 0.05 and a delta score between two spliced peptide hits or between a top scoring spliced peptide and a lower scoring non-spliced peptide of 30% (12). Mascot Distiller's label-free quantification toolbox was used to automatically extract MS ion peak areas of all identified peptides for all five time points (0–4 h) and all three technical replicates simultaneously. Biological replicates were processed separately. The resulting peptide kinetics were filtered for peptide synthesis artifacts and non-reproducible peptide kinetics between technical replicates. Furthermore, peptides that showed unrealistic generation kinetic behavior (such as alternating MS ion peak areas between consecutive time points) were removed. In the final analysis, only peptides that were detected and quantified in two biological replicates were considered.

KRAS_5−6/8−14_ G12V and KRAS_5−14_ G12V generation kinetics were manually quantified by extraction of an ion chromatogram (XIC) corresponding to the peptides monoisotopic peaks, using instrument precursor tolerance and retention time information (from the identified peptides in the 20 h digestions) via Mascot Distiller, followed by determination of the area under the peak at each time point in the kinetics series.

Absolute peptide quantification was carried out through the application of the method QPuB on detected MS ion peak areas for each peptide product, as described elsewhere (see Data availability section). In the specific case of the two epitope candidates KRAS_5−6/8−14_ G12V and KRAS_5−14_ G12V, we computed their amount using a titration curve of the cognate synthetic peptides since their amount was too low to be estimated with high confidence using QPuB. Synthetic peptide concentration for titration ranged from 0 to 10 pmol injected. Each titration sample was measured twice and right after measuring *in vitro* digestion samples.

SCS-P1 (site specific cleavage strength after amino acid residue P1) and PSP-P1 (frequency of peptide splicing catalyzed using the C-terminus of the N-terminal splice-reactant as splicing site) were calculated based on the absolute amount of each product (resulting from QPuB) identified in the proteasome-catalyzed digestions (23). Briefly, for each time point and each amino acid in the substrate, the sum over all product (non-spliced and spliced peptides) amount that have the corresponding substrate amino acid at their C-terminus has been computed and normalized, so that they add up to 100%, resulting in SCS-P1. For each time point and each amino acid in the substrate, the sum over all spliced peptide amount that have the corresponding substrate amino acid at their C-terminus of the N-terminal splice-reactant was computed and normalized, so that they add up to 100%, resulting in PSP-P1.

### TAP Assay

The transport efficiency of target peptides (Supplementary Table 2) into the ER lumen mediated by TAPs was carried out as previously described (29) although some modifications were introduced. These include the use of a fluorescent peptide tracer and the use of microsomes rather than Streptolysin O permeabilized cells. Briefly, peptides were dissolved in DMSO and different concentrations were distributed in a final volume of 10 μl DMSO. At the same time, a mixture of 10 mM ATP, 100 mM Tris-HCl pH7.5 and 5 mM MgCl_2_ and fluorescent tracer peptide was prepared. 60 μl of this mixture was added to the 10 μl competing peptide mixture to a final volume of 70 μl. This was prewarmed to 37°C and 30 μl of pre-warmed microsomes were added. Microsomes were derived from LCL721 cells, as previously described (29).

The mixture was incubated for 20 min at 37°C followed by cell lysis with lysis mixture (0.5% TX100, 5 mM MgCl_2_ in 100 mM Tris-HCl pH7.5) at 4°C. After at least 30 min incubation at 4°C, DNA was pelleted and the supernatant transferred to a new vial including ConA-beads. After at least 30 min incubation on ice, cells were washed four times with lysis mixture and the last time with 100 mM Tris-HCl pH7.5 before transfer to 96 wells plates (Corning) followed by fluorescence measurements in a plate reader. To detect background signals, a sample without competing peptide and ATP was included and fully performed as described above. This signal was subtracted from the detected signal. The curves were normalized to the highest value set at 100% and EC_50_ values were calculated.

### HLA-I–Peptide Binding Affinity Prediction and Measurement

The binding affinity between theoretical spliced and non-spliced peptides and HLA-A^*^02:01 was predicted using the NetMHCPan 3.0 algorithm (30). We restricted the prediction to 8–12 mer peptides and imposed an IC_50_ cut-off of 100 nM. The binding affinity between the synthetic peptides and HLA-A^*^02:01 complexes was measured using purified HLA-I molecules, as described elsewhere (10).

### HLA-I-Peptide Crystal Structure and Analysis

The ectodomains of HLA-A^*^02:01 (residues 21–274) and human β_2_-microglobulin (hβ_2_m) (residues 1–99) were expressed in *Escherichia coli* BL21 DE3 cells as inclusion bodies after 4 h induction with 1 mm isopropyl 1-thio-d-galactopyranoside (IPTG) at *A*_600_ of 0.6. Cells were harvested by centrifugation (5,000 × *g* for 20 min), resuspended in lysis buffer (100 mM Tris-HCl, pH7.0, 5 mM EDTA, 5 mM DTT, 0.5 mM PMSF), and broken through a microfluidizer (Microfluidics). Inclusion bodies were collected from cell lysate (50,000 × *g* for 30 min at 4°C), washed 3 times in 100 mM Tris-HCl, pH7.0, 5 mM EDTA, 5 mM DTT, 2 M urea, 2% (w/v) Triton X-100 plus 1 time in 100 mM Tris-HCl, pH7.0, 5 mM EDTA, 2 mM DTT), and finally dissolved in 50 mM Tris-HCl, pH7.0, 5 mM EDTA, 2 mM DTT, 6 M guanidine HCl) for the following refolding.

Refolding was performed in a 100 ml system. Briefly, 1.2 mg of hβ_2_m was loaded dropwise into refolding buffer (0.1 M Tris-HCl, pH8.0, 2 mM EDTA, 400 mM l-arginine, 5 mM oxidized glutathione, 5 mM reduced glutathione) and stirred for 1 h at 4°C. Then, 6 mg of HLA-A^*^02:01 mixed with 1.2 mg of individual peptide (Supplementary Table 2) was added dropwise into the refolding system and stirred at 4°C for up to 72 h. The refolding system was concentrated to 0.5 mL for size exclusion chromatography using a Superdex S200 Increase 10/300 GL column in 20 mM Tris-HCl pH7.5, 150 mM NaCl. Fractions containing refolded HLA-A^*^02:01-β_2_m-peptide complexes were pooled and concentrated to 5–10 mg/ml for subsequent crystallization.

Thick plate-like or 3-dimensional crystals of HLA-A^*^02:01-β_2_m-peptide complexes were obtained by setting drop vapor diffusion at 1:1–1.5 ratio with 30% PEG 4000, 0.1 M Tris-HCl, pH8.5, 0.2 M lithium sulfate at room temperature after 3 days. The crystals were flash frozen in crystallization solution plus glycerol (25% v/v) using liquid nitrogen.

Diffraction data for HLA-A^*^02:01-β_2_m-peptides were collected remotely at beam line 9.2 at the Stanford Synchrotron Radiation Light source and processed to 1.40–1.55 Å resolution, using HKL2000. Phases were obtained by molecular replacement with Phaser MR in ccp4 using the protein coordinates from a former HLA-A^*^02:01-β_2_m-peptide structure (Protein Data Bank code 5ENW) (31). The model was built with COOT (32) and refined with REFMAC5 (33). Data collection and refinement statistics are shown in Supplementary Table 3.

### Statistical Analysis

If not stated otherwise, all statistical tests have been done in R and differences in distributions have been tested using the Kolmogorov-Smirnov test.

### Dataset and Software Availability

A summary of the RAW files of the LC-MS/MS measurements of the *in vitro* digestions accessible via repository is reported in the following Mendeley dataset: http://doi.org/10.17632/63rj3xczmb.1.

The mass spectrometry proteomics data have been deposited to the ProteomeXchange Consortium via the PRIDE (34) partner repository with the dataset identifier PXD015580.

The HLA-I immunopeptidome elution MS files used in the first step of the pipeline are available at the PRIDE repository with the dataset identifier PXD000394 (files: 20120321_EXQ1_MiBa_SA_HCC1143_1.raw, 20120321_EXQ1_MiBa_SA_HCC1143_2.raw, 20120322_EXQ1_MiBa_SA_HCC1143_1_A.raw, 20120515_EXQ3_MiBa_SA_HCT116_mHLA-1.raw, 20120515_EXQ3_MiBa_SA_HCT116_mHLA-2.raw, 20120617_EXQ0_MiBa_SA_HCT116_1_mHLA_2hr.raw, 20120617_EXQ0_MiBa_SA_HCT116_2_mHLA_2hr.raw) and at the Datadryad.org archive (doi: 10.5061/dryad.r984n) and were generated by Bassani et al. (35) and Mommen et al. (36).

QPuB software is available at GitHub (https://github.com/QuantSysBio/QPuB).

## Results

### Prioritization of KRAS as Antigen Over-represented on Cell Surface

Antigens represented by spliced peptides in HLA-I immunopeptidomes tend to be preferentially long, hydrophobic and basic, thereby suggesting that the chemical and physical characteristics of antigens can impinge upon spliced peptide generation and presentation (12, 24). To select suitable antigens from which a spliced epitope candidate might be derived, we first investigated which combination of protein features may result in more likely potential over-representation in HLA-I immunopeptidomes by spliced and non-spliced peptides.

Accordingly, we used a previously published HLA-I spliced and non-spliced immunopeptidome database (12), which includes 13,666 unique non-spliced and 1,318 unique spliced peptides, as well as 7,328 represented antigens. With this dataset, we developed a simple model based on protein length, hydrophobicity, isoelectric point and instability index to determine the possible over-representation of a given antigen by spliced and non-spliced peptides in HLA-I immunopeptidomes. These characteristics were previously described to influence the probability of observing peptides of a protein being presented in HLA-I immunopeptidomes determined by antigen gene expression level and antigen abundance as key determinants for efficient presentation (12, 37). However, we here opted to focus on protein intrinsic characteristics that are conserved independently of cell types and cell status, to obtain a model for antigen selection that can be generalized.

**Figure 2:**
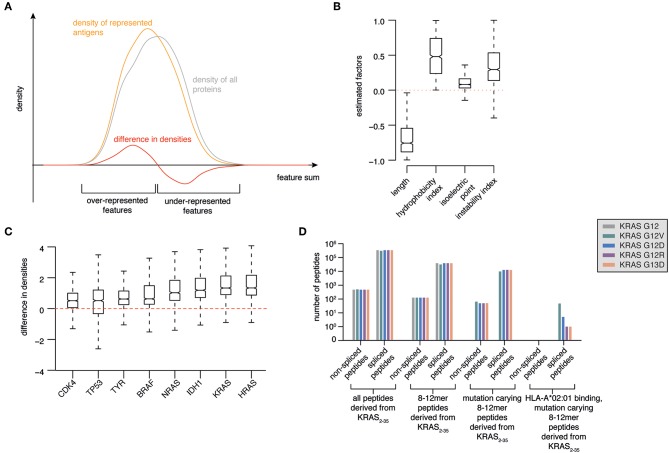
Prioritization of KRAS G12V as source of potentially antigenic HLA-A*02:01-bound spliced peptides. **(A)** Illustration of a model to select antigens with features more likely to be over-represented in HLA-I immunopeptidomes. Protein length, hydrophobicity, isoelectric point and instability are used to calculate a protein feature sum for all represented antigens. The resulting distribution (orange line) is compared to the feature sum distribution based on all human proteins (gray line). The difference between these two distributions (red line) indicates which features favor and disfavor the representation of antigens in HLA-I immunopeptidomes. **(B)** Marginal posterior distributions of the estimated factors that maximize the difference between feature sum distribution of represented antigens vs. all proteins. **(C)** Ranking of selected tumor-associated antigens BRAF, KRAS, HRAS, NRAS, TP53, CDK4, IDH1, TYR as antigens with features over-represented in HLA-I immunopeptidomes. **(D)** Progressive reduction of the theoretical number of spliced and non-spliced epitope candidates, thereby narrowing down to those carrying KRAS G12V/D/R or G13D mutations (*in silico* derived from the KRAS_2−35_ sequence) and predicted to bind HLA-A*02:01 with IC_50_ ≤ 100 nM.

Combining these four selected protein characteristics yielded a distribution of known represented antigens, which can be compared to the feature distribution of all proteins (Figure 2A). Proteins with feature values that show a higher density for represented antigens compared to all proteins are more likely to be favored for antigen presentation than those proteins with feature values that show a lower density for represented antigens compared to all proteins (Figure 2A). We therefore aimed to find a combination of features that maximizes the difference between the two distributions. We defined a model calculating a feature sum ∑*p*_*i*_ *c*_*i*_, where *p*_*i*_ are factors ranging from −1 (favoring representation) to 1 (disfavoring representation) and *c*_*i*_ are the protein characteristic values.

Using Bayesian inference, we estimated the factors that provide the largest distance between the resulting feature sum distributions for represented antigens compared to all proteins. We found that protein length favors representation. On the contrary, very hydrophobic or instable proteins are disfavored during representation. The isoelectric point appeared to have minor influence (Figure 2B).

As proof-of-principle, we focused our analysis on a series of major tumor antigens - BRAF, KRAS, HRAS, NRAS, TP53, CDK4, IDH1, TYR - which all carry recurrent oncogenic mutations. We calculated the feature sums for those eight antigens and determined corresponding density differences for each of those feature sums, which allowed us to rank the candidate antigens (Figure 2C). Among them, HRAS and KRAS are the two antigens that are most likely over-represented in HLA-I immunopeptidomes as compared to the whole proteome (Figure 2C).

### Prioritization of KRAS G12V neoantigen as Source of Potentially Antigenic HLA-A^*^02:01-Bound Tumor-specific Spliced Peptides

HRAS and KRAS are two GTPases that function as molecular switches in regulatory pathways responsible for proliferation and survival. In particular, KRAS is frequently mutated in cancers with an average of 22% cancers carrying a KRAS mutation, a frequency that rises to 33–61% in colorectal cancer and pancreatic adenocarcinoma (38). The mutations often occur in the KRAS G12 and G13 residues, which impairs the KRAS GTPase activity and renders the mutants persistently in the GTP-bound active form, thereby promoting tumorigenesis and tumor malignancy (39). KRAS G12/13 is a driver in tumors and in combination with its high frequency in cancers makes KRAS an ideal target for immunotherapies. For example, ATT using multiple T cell Receptors (TCRs), which recognize HLA-A^*^02:01^+^ spliced epitopes carrying KRAS mutations, could treat around 30% of pancreatic adenocarcinoma patients and a large portion of colorectal carcinoma patients. The efficacy of ATT has been demonstrated in a xenograft mouse model (40) and a metastatic colorectal cancer patient (41) by targeting non-spliced epitopes carrying KRAS G12D mutations and presented by HLA-A^*^11:01 or -C^*^08:02 molecules, respectively.

All these features define KRAS as an attractive tumor antigen to be further investigated using our pipeline. We investigated *in silico* the sequence surrounding residues 12 and 13 of KRAS wild type, G12V/D/R and G13D antigens. All spliced and non-spliced peptides that could be theoretically generated were computed. From this list, we removed all peptide candidates not carrying the target mutations, as well as those candidates shorter than 8 residues or longer than 12 residues, which is the length range most often observed in HLA-I immunopeptidomes (Figure 2D). Since the HLA-A^*^02:01 allele is the predominant allele in Caucasian populations, we predicted the binding affinity of this HLA-I variant to the remaining peptides using the NetMHCPan 3.0 algorithm (30). Finally, we filtered out all peptides that were predicted to bind with IC_50_ > 100 nM. None of the non-spliced epitope candidates passed this step, whilst 54 spliced epitope candidates had the required features. Among them, 47 can theoretically carry the KRAS G12V mutation (Figure 2D).

### Identification of KRAS G12V^+^ Spliced Epitope Candidates Generated by Proteasomes

**Figure 3:**
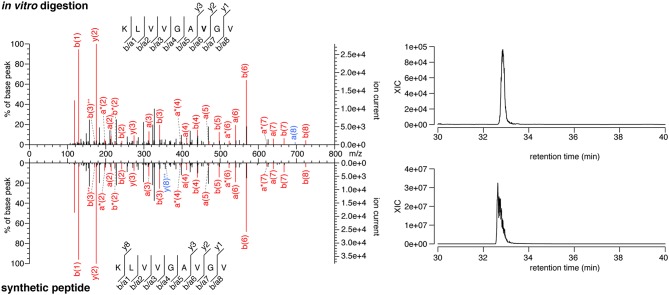
MS/MS spectra of the KRAS_5−6/8−14_ G12V spliced epitope candidate. MS/MS spectrum of the peptide KRAS_5−6/8−14_ G12V [KL][VVGA**V**GV] identified in the *in vitro* digestion of the synthetic polypeptide KRAS_2−35_ G12V and the MS/MS spectrum of the cognate synthetic peptide (left panels). The peptide sequence is shown with the corresponding b-, a- and y-ions identified. The G12V mutation is depicted in bold. In the spectra, assigned peaks for b-, a-, and y-ions are reported in color. Ion neutral loss of ammonia is symbolized by *. Red marked peaks are assigned in both *in vitro* digestion detected MS/MS spectrum and synthetic peptide MS/MS spectrum, whereas blue marked peaks are assigned only in one of the two spectra. The extracted ion chromatogram for the peptide identified in the *in vitro* digestion and the synthetic counterpart is plotted and indicates matching retention times for both peptides (right panels).

The majority of the HLA-I-restricted epitopes are produced by proteasomes. Their production can be verified through *in vitro* digestion of synthetic polypeptides by 20S proteasomes, as measured by MS. Because of the high frequency of putative HLA-A^*^02:01^+^ spliced epitope candidates carrying the KRAS G12V mutation (KRAS G12V^+^), we focused on this mutation and digested the synthetic KRAS_2−35_ wild type and G12V polypeptides with 20S standard proteasomes for 20 h. The digestions were measured by targeted MS, which used a m/z inclusion list of target spliced epitope candidates identified in the previous pipeline step (Supplementary Table 1), and confirmed that one spliced epitope candidate, KRAS_5−6/8−14_ G12V [KL][VVGA**V**GV], is generated by proteasomes under these conditions (Figure 3). This spliced peptide could be generated by the removal of one of the three V residues in the KRAS sequence, i.e., it could be reported as KRAS_5−6/8−14_ G12V, KRAS_5−7/9−14_ G12V, or KRAS_5−8/10−14_ G12V. We will refer to it as KRAS_5−6/8−14_ G12V for the sake of simplicity.

Notably, this spliced peptide is not present in the reaction at *t* = 0 and the 20 h reaction containing the synthetic polypeptide substrate in absence of proteasomes (data not shown). The cognate spliced peptide KRAS_5−6/8−14_ G12 [KL][VVGA**G**GV] is not produced by 20S proteasomes whilst processing the synthetic wild type KRAS_2−35_ polypeptide.

In the KRAS_2−35_ G12V polypeptide digestion, we also identified the non-spliced epitope candidate KRAS_5−14_ G12V [KLVVVGA**V**GV] (Supplementary Figure 1). The spontaneous response of peripheral blood mononuclear cells (PBMCs) of pancreatic adenocarcinoma patients against this latter epitope candidate was previously described (42). Although this peptide was filtered out in the early steps of our pipeline because it has a predicted HLA-A^*^02:01 binding affinity IC_50_ > 100 nM, we compared this epitope candidate to the KRAS_5−6/8−14_ G12V spliced epitope candidate in the next validation steps.

### Spliced Peptide and KRAS_5–6/8–14_ G12V Spliced Epitope Candidate Production Kinetics by Proteasomes

To be a robust epitope candidate, a peptide should be produced *in vitro* by proteasomes in a detectable amount and with consistent kinetics. Correspondence between *in vitro* experiments carried out with purified 20S proteasomes and *in cellulo* and *in vivo* experiments has been demonstrated in various studies investigating both viral and tumor epitopes (4, 5, 9, 17, 19, 21, 43–50). Therefore, we performed digestion kinetics (0–4 h) of the synthetic KRAS_2−35_ wild type and G12V polypeptides with 20S standard proteasomes. The samples were measured by MS to identify all digestion products (via MS/MS). Quantification of peptides was performed using QPuB, a method that uses detected MS ion peak areas to estimate the absolute amount of each spliced and non-spliced peptide products (see Data Availability section), and by comparison with synthetic peptide titration for the two epitope candidates.

**Figure 4:**
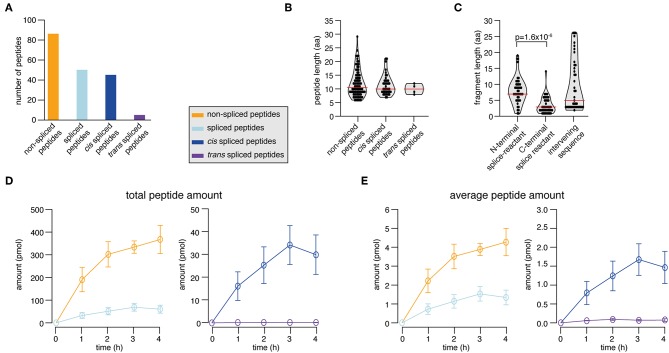
Spliced peptide characteristics and kinetics in KRAS_2−35_ G12V polypeptide degradation. **(A–D)** Results of the analysis of *in vitro* digestions of the synthetic KRAS_2−35_ G12V polypeptide substrate by 20S proteasomes (two biological replicates each measured three times). Spliced and non-spliced peptide products were identified by MS and quantified by applying QPuB. Only peptides identified in both biological replicates with reproducible kinetics have been analyzed. **(A)** Number of non-spliced, *cis* spliced, and *trans* spliced peptides identified in the reactions. **(B)** Length distribution of non-spliced peptides, *cis* spliced and *trans* spliced peptides. **(C)** Length distribution of N- and C-terminal splice-reactants of *cis* spliced peptides as well as of their intervening sequences. **(D)** Total amount of spliced and non-spliced, as well as *cis* spliced and *trans* spliced peptides quantified by applying QPuB to *in vitro* kinetics. **(E)** Abundance of an average spliced and non-spliced, as well as *cis* spliced or *trans* spliced, peptides in the *in vitro* kinetics over the digestion time. In **(B,C)**, violin plots indicate the fragment length distribution. Red lines indicate the median. When statistically significant, *p-*values are reported.

In the synthetic KRAS_2−35_ G12V polypeptide digestion, we identified and successfully quantified 131 peptide products. 65.6% were non-spliced, 31.3% *cis* spliced and 3.1% *trans* spliced peptides (Figure 4A). The length distribution of the non-spliced, *cis* spliced and *trans* spliced peptides did not significantly differ and its median was 10 amino acid residues (Figure 4B). N- and C-terminal splice-reactants had a median length of 7 and 3 amino acid residues, respectively (Figure 4C). The intervening sequences of *cis* spliced peptides had a median length of 5 amino acid residues (Figure 4C). From the quantitative point of view, *cis* and *trans* spliced peptides represent proximately 17.0 and 0.1% of the peptide abundance, respectively (Figure 4D). On average, a *trans* spliced peptide is less abundant than a *cis* spliced peptide, which is less abundant than an average non-spliced peptide (Figure 4E).

**Figure 5:**
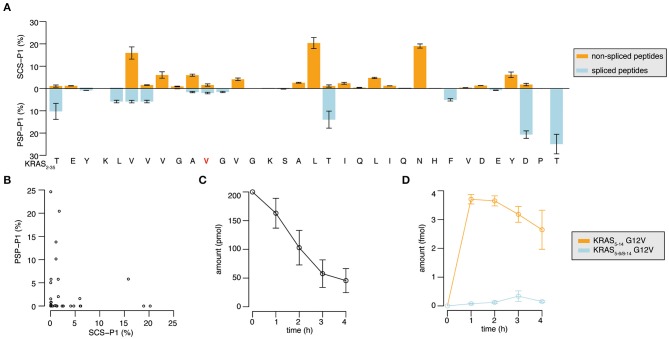
Substrate cleavage- and splicing-site preferences and generation kinetics of the KRAS_5−6/8−14_ G12V and KRAS_5−14_ G12V epitope candidates. **(A)** Relative usage of the substrate sites for cleavage (SCS-P1), and splicing (PSP-P1) in the *in vitro* digestion kinetics of the synthetic KRAS_2−35_ G12V polypeptide substrate by 20S proteasomes (two biological replicates each measured three times). Mean and *SD* of biological replicates (bars) are shown. **(B)** Scatter plot of SCS-P1 and PSP-P1, which depicts the absence of direct correlation between splicing and cleavage frequencies. In **(A,B)** spliced and non-spliced peptide products were identified by MS/MS in the *in vitro* digestion kinetics of synthetic polypeptide KRAS_2−35_ G12V with 20S proteasomes, and were quantified by applying QPuB based on their MS ion peak area. SCS-P1 and PSP-P1 were computed using the average amount of all time points of each peptide product. **(C,D)** Abundance of the synthetic polypeptide substrate KRAS_2−35_ **(C)** as well as the KRAS_5−6/8−14_ G12V and KRAS_5−14_ G12V epitope candidates **(D)** in the *in vitro* digestions with 20S proteasomes (three biological replicates each measured 3–4 times). Spliced and non-spliced peptide products were identified by MS/MS and quantified based on their MS ion peak area, using titration of synthetic peptides as reference.

Through the application of QPuB to the synthetic KRAS_2−35_ wild type and G12V polypeptide digestions, we could also compute how frequently proteasomes cleaved the substrate after each of its individual residues (substrate cleavage strength, i.e., SCS-P1) or used each residue for the PCPS reaction (proteasome-generated spliced peptide P1 positions, i.e., PSP-P1). From this analysis we confirmed our previous hypothesis (23), whereby proteasomes splice at sites at which the substrates are less frequently cleaved at (and *vice versa*), as emerged by comparing SCS-P1 and PSP-P1 (Figures 5A,B).

The quantitative analysis of the KRAS_2−35_ synthetic substrate degradation (Figure 5C) also showed that the KRAS_5−6/8−14_ G12V spliced epitope candidate is produced in amounts smaller than the average amount of spliced peptides (Figures 4E, 5D shall be compared).

### KRAS_5–6/8–14_ G12V Spliced Epitope Candidate Is a TAP Substrate and Efficiently Binds Hla-A^*^02:01

**Figure 6:**
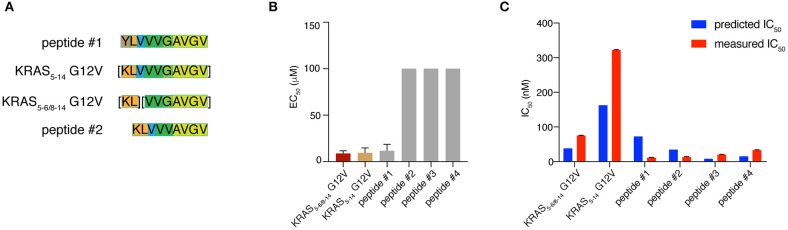
KRAS_5−6/8−14_ G12V epitope candidate is efficiently transported by TAPs and strongly binds HLA-A*02:01 complex. **(A)** Sequence comparison between KRAS_5−6/8−14_ G12V [KL][VVGA**V**GV] and KRAS_5−14_ G12V [KLVVVGA**V**GV] epitope candidates as well as their modified versions (peptides #1 YLVVVGAVGV and #2 KLVVVAVGV). Common sequences among peptides are color-coded. **(B)** Transport efficiency into the ER lumen mediated by TAPs of KRAS_5−6/8−14_ G12V and KRAS_5−14_ G12V epitope candidates, their modified versions (peptides #1 and #2) and two unrelated peptides (peptides #3 and #4; Supplementary Table 2). The EC_50_ was computed using a competing peptide as reference. We here report the EC_50_ values obtained upon subtracting the peptide transport in absence of ATP. **(C)** Predicted and measured binding affinities of the peptides to the HLA-A*02:01 complex. Binding affinity prediction was carried out with the NetMHCPan 3.0 algorithm. In **(B,C)** mean and SD of biological replicates (bars) are shown.

The production of a peptide by proteasomes is not sufficient alone to incur presentation on the cell surface. There are several other steps in the APP pathway that can direct the peptide fate, such as peptide transport into the ER lumen mediated by TAPs and peptide binding to HLA-A^*^02:01 complex. We studied the KRAS_5−6/8−14_ G12V [KL][VVGA**V**GV] spliced epitope candidate in comparison with the KRAS_5−14_ G12V [KLVVVGA**V**GV] non-spliced epitope candidate. We also extended the study to four control peptides. Two peptides - peptide #1 YLVVVGAVGV and peptide #2 KLVVVAVGV - shared a large portion of the KRAS_5−6/8−14_ G12V and KRAS_5−14_ G12V epitope candidate sequences (Figure 6A). The other two control peptides are unrelated peptides predicted to bind to the HLA-A^*^02:01 complex (peptides #3 FLHEDLEKI and #4 FLHEDTEKI; see Supplementary Table 2).

To quantify the efficiency of peptide transport into the ER lumen by TAP, we measured the competition between the target peptides and a fluorescent reference peptide for TAP-dependent translocation into free microsomes. The reference peptide has an N-linked glycosylation consensus sequence and peptide glycosylation is used to monitor entry into ER microsomes and as an isolation handle (29). KRAS_5−6/8−14_ G12V epitope candidate is efficiently transported by TAPs as the KRAS_5−14_ G12V non-spliced epitope candidate is. Peptide #1, which has a K to Y substitution at position 1 as compared to KRAS_5−14_ G12V peptide, is transported by TAP as efficiently as the non-spliced epitope candidate. In contrast, peptide #2, which has the removal of residue G in position 6 as compared to KRAS_5−14_ G12V peptide and a G to V substitution at position 5 as compared to KRAS_5−6/8−14_ G12V peptide, is not competing with the reference peptide and thus ignored by TAP. This suggests a role of the residue G at the center of the peptides in TAP-mediated transport. The other two control peptides are not substrates for TAP (Figure 6B).

Once a peptide arrives in the ER lumen, its binding affinity to the specific HLA-I molecule determines whether it will ultimately be presented. Therefore, we measured the binding affinity of the same six peptides previously tested in the TAP assay and the HLA-A^*^02:01 complex in a cell-free system utilizing purified HLA-I molecules. The KRAS_5−6/8−14_ G12V epitope candidate was confirmed to efficiently bind the HLA-A^*^02:01 complex, in contrast to the KRAS_5−14_ G12V non-spliced epitope candidate, which had a measured IC_50_ larger than 300 nM (and larger than predicted). The measured and predicted IC_50_ of the control peptides was quite similar and the peptides appear to be good binders (Figure 6C).

### Conformation of KRAS_5–6/8–14_ G12V and KRAS_5−14_ G12V Epitope Candidates Within HLA-A^*^02:01 cleft

**Figure 7:**
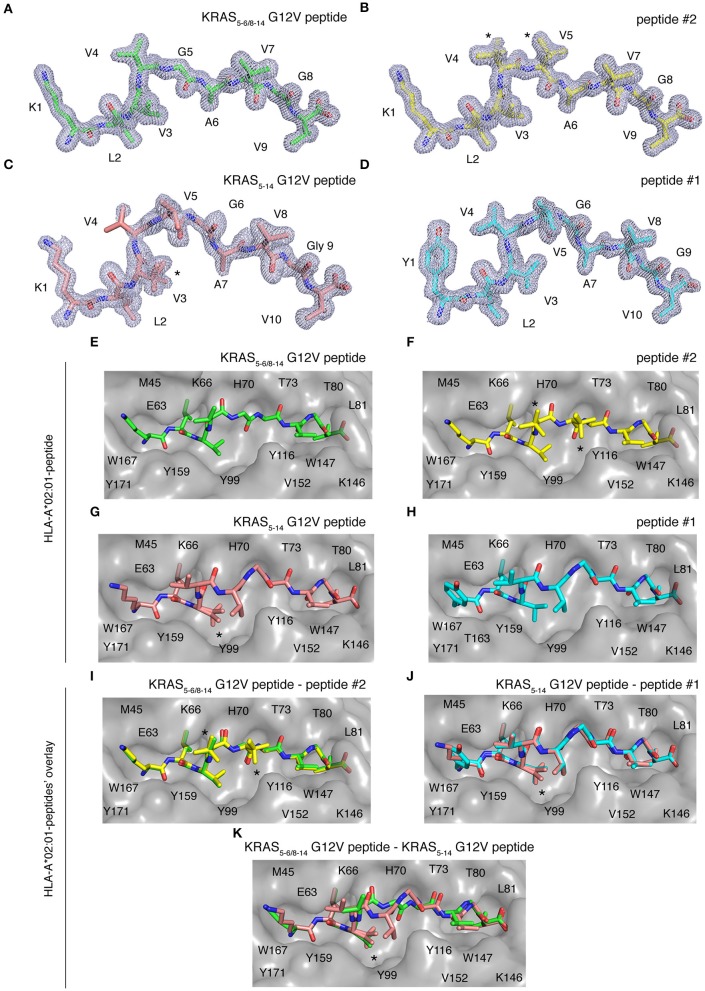
HLA-A*02:01-KRAS G12V peptides binding mode. Binding mode of spliced epitope candidate KRAS_5−6/8−14_ G12V [KL][VVGAVGV], non-spliced epitope candidate KRAS_5−14_ G12V [KLVVVGAVGV], peptides #1 YLVVVGAVGV and #2 KLVVVAVGV to HLA-A*02:01 complex. **(A–D)** 2Fo-Fc electron density map contoured at 1σ for KRAS_5−6/8−14_ G12V peptide **(A)**, peptide #2 **(B)**, non-spliced peptide KRAS_5−14_ G12V **(C)**, and peptide #1 **(D)**. **(E–H)** Binding of spliced peptide KRAS_5−6/8−14_ G12V (**E**; green sticks), peptide #2 (**F**; yellow sticks), non-spliced peptide KRAS_5−14_ G12V (**G**; brown sticks), and peptide #1 (**H**; cyan sticks) to HLA-A*02:01 protein (gray molecular surface). **(I–K)** Overlay of KRAS_5−6/8−14_ G12V and peptide #2 binding to HLA-A*02:01 **(I)**, KRAS_5−14_ G12V peptide and peptide #1 **(J)**, as well as KRAS_5−6/8−14_ G12V and KRAS_5−14_ G12V peptides **(K)** binding to HLA-A*02:01 molecule. All peptides are shown as sticks and in **(A–D)**, peptide residues are labeled with one-letter amino acid codes. In **(E–K)**, the residues of HLA-A*02:01 that are extended in the peptide binding interface are labeled with single-letter amino acid codes. In **(B,F,I)**, * indicates the alternate conformations for residues V4 and V5 of peptide #2. In **(C,G,J–K)**, *indicates the alternate conformations for residues V3 of KRAS_5−14_ G12V peptide.

Once a peptide is bound to HLA-I complexes and presented at the cell surface, it can be recognized by TCRs of CD8^+^ T cells. The conformation of the peptide in the HLA-I groove is paramount not only for HLA-I-peptide affinity and stability, but also for the TCR-HLA-I-peptide interaction. To study this aspect, we individually refolded and crystallized HLA-A^*^02:01 with spliced epitope candidate KRAS_5−6/8−14_ G12V, non-spliced epitope candidate KRAS_5−14_ G12V, as well as two control peptides (peptides #1 and #2), in which either the N-terminal residue or one of the central residues was substituted, as compared to the epitope candidates (Figures 7A–K). The crystal structures of these individual HLA-I-peptide complexes were determined at resolutions ranging from 1.4 to 1.58 Å by molecular replacement using PDB ID 5ENW as a search model (Supplementary Table 3). The global superposition of all these four peptide-HLA-I complexes in the peptide binding groove reveals a similar binding orientation with a root mean square deviation value (rmsd) of 0.124 Å. The electron densities for all four peptides are also well-defined over the entire peptide length.

Some structural differences in individual peptide binding were observed when comparing the 9 mer peptides (KRAS_5−6/8−14_ G12V [KL][VVGA**V**GV] and peptide #2 KLVVVAVGV) with the 10 mer peptides (KRAS_5−14_ G12V [KLVVVGA**V**GV] and peptide #1 YLVVVGAVGV). Comparison of peptides with a same length generally only reveals a single amino acid change in a similar orientation or the addition of a side chain, e.g., when V replaces G in peptide #2 as compared to KRAS_5−14_ G12V peptide (Figures 7A–D). Specifically, while the N-terminal and C-terminal ends of all four peptides match perfectly, structural superposition reveals conformations in the middle portions of peptide KRAS_5−6/8−14_ G12V and peptide #2 unique from the remaining two peptide ligands (KRAS_5−14_ G12V and peptide #1). In the latter two cases, the middle portions of the peptides containing P4, P5, and P6 residues bulge out of the binding pockets to accommodate both peptide ends inside the peptide-binding groove of the HLA-I molecule (Figures 7C,D,G,H).

We next evaluated at the detailed interactions between HLA-A^*^02:01 and individual peptides. Throughout these interfaces, extensive hydrophobic and hydrogen bonding networking is seen with the majority of peptide residues participating in the contact with HLA-I residues Y7, F9, M45, E63, K66, V67, H70, T73, T80, L81, Y84, Y99, Y116, T143, K146, W147, V152, Y159, W167, Y171 (Figures 7E–H).

In the HLA-I-peptide #1 complex, the N-terminal P1 Y residue makes hydrophobic contact with T163, which is missing in all the other three peptide complexes and may explain the significantly higher binding affinity of 10 mer peptide #1 compared to 10 mer KRAS_5−14_ G12V peptide (Figure 7H). The A and F pockets forming the peptide binding groove of HLA-I are mostly composed of hydrophobic residues and some 12 polar and 21 van der Waals contacts were, throughout the peptide length in all complexes, observed between the peptide moiety and HLA-A^*^02:01.

While the HLA-I interaction interface seems to be conserved in both spliced peptide KRAS_5−6/8−14_ G12V and non-spliced peptide KRAS_5−14_ G12V, the binding affinity of KRAS_5−6/8−14_ G12V peptide toward HLA-A^*^02:01 is higher compared to the KRAS_5−14_ G12V peptide (Figure 6C). Hence, to understand this differential affinity of these peptides for HLA-A^*^02:01, we compared the crystal structures of KRAS_5−6/8−14_ G12V and KRAS_5−14_ G12V peptides and modified variants (peptide #1 and #2) bound to HLA-A^*^02:01 complexes in more detail (Figures 7I–K). The superpositions of either KRAS_5−6/8−14_ G12V peptide and peptide #2 or KRAS_5−14_ G12V peptide and peptide #1 do not show any relevant differences (Figures 7I,J).

In contrast, although the structural superposition of KRAS_5−6/8−14_ G12V and KRAS_5−14_ G12V peptides bound to HLA-A^*^02:01 molecules reveals a similar type of HLA-I interaction network at their N-terminal and C-terminal regions, their structural arrangements deviate in their middle portions. Due to this, the spliced peptide KRAS_5−6/8−14_ G12V makes several unique interactions with HLA-A^*^02:01. Firstly, in the structure of HLA-A^*^02:01 complexed with spliced peptide KRAS_5−6/8−14_ G12V, the P6 A residue makes both hydrogen bonding and van der Waals contacts with the side chain of T73 residue of HLA-I, whereas the P6 G residue of KRAS_5−14_ G12V is not in contact with HLA-A^*^02:01 and its P7 A residue maintains only hydrophobic interactions with T73.

Another difference between both these complexes is at their C-termini. In the HLA-I-KRAS_5−14_ G12V peptide complex, the HLA-A^*^02:01 K146 residue adopts a different orientation, due to which it interacts with only the terminal PΩ residue. In the HLA-I-KRAS_5−6/8−14_ G12V complex, the amino group of K146 forms a hydrogen bond with both the carbonyl oxygen of the PΩ-1 residue and the terminal PΩ residue. Furthermore, though KRAS_5−14_ G12V is longer [10 amino acids, compared to KRAS_5−6/8−14_ G12V (9 amino acids)] and reorganizes its central region in the HLA binding groove, this structural rearrangement does not favor any additional contacts with HLA-A^*^02:01. From our structural analysis, we can predict that the higher affinity of the spliced KRAS_5−6/8−14_ G12V peptide, compared to KRAS_5−14_ G12V peptide, might be due to these two additional hydrogen bonding contacts between the spliced peptides P6 A residue and the T73 of HLA-A^*^02:01, as well as the spliced peptides PΩ-1 residue and the K146 residue of the HLA-A^*^02:01 molecule (Figure 7K).

### Potential Recognition of KRAS_5–6/8–14_ G12V and KRAS_5−14_ G12V Epitope Candidates Within HLA-A^*^02:01 Cleft by Different TCRs

**Figure 8:**
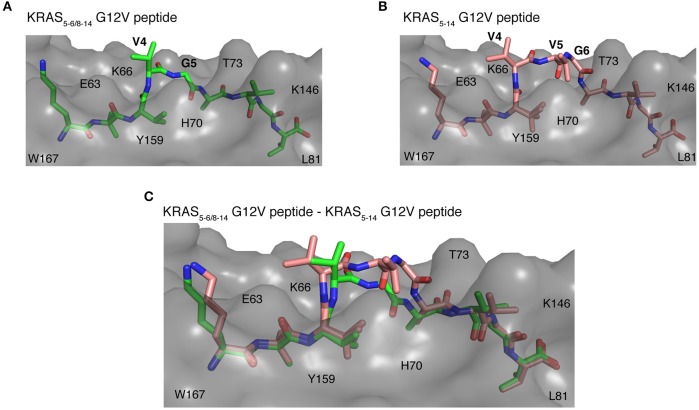
Potential recognition of KRAS_5−6/8−14_ G12V and KRAS_5−14_ G12V epitope candidates within HLA-A*02:01 cleft by different TCRs. **(A,B)** Side view of binding mode of spliced epitope candidate KRAS_5−6/8−14_ G12V [KL][VVGAVGV] (**A**; green sticks), non-spliced epitope candidate KRAS_5−14_ G12V [KLVVVGAVGV] (**B**; brown sticks) into the HLA-A*02:01 binding grove displaying the peptide residues exposed for TCR recognition. **(C)** Structural superposition of KRAS_5−6/8−14_ G12V and KRAS_5−14_ G12V peptides complexed with HLA-A*02:01 molecule revealing the conformational deviation at their middle region where TCR interaction is expected to happen. In all three panels, the HLA-A*02:01 protein is shown as gray molecular surface and peptide residues as sticks. In **(A,B)**, peptide residues exposed for TCR recognition are labeled. In all panels, some of the residues of HLA-A*02:01 that are extended in the peptide binding interface are labeled.

Once the peptide binds to a HLA-I molecule, it gets displayed for TCR recognition, which can then induce effective immune responses. Using structure as a tool, we tried to determine the mode of TCR-HLA-I-peptide interaction. Our evaluation of HLA-I-KRAS_5−6/8−14_ G12V and HLA-I-KRAS_5−14_ G12V peptide complexes provides a link to the potential cross recognition by a given CD8^+^ T cell clone. The middle portion of both spliced and non-spliced epitope candidates containing P4 and P5 residues does not make ample contacts with the HLA-A^*^02:01 molecule but has limited flexibility in the crystal structure, otherwise this would not have been solved in the structure (Figures 8A,B). In the HLA-A^*^02:01-KRAS_5−14_ G12V peptide complex, the middle portion that bulges out from the binding groove makes it more accessible for TCR recognition (Figure 8B). The side chain of P4 V and P5 V residues are facing in an upward direction and can be easily accommodated into the binding pocket located over the central peptide, formed by the most structurally diverse CDR loops, CDR3α and CDR3β of the TCR. Similarly, in the HLA-I-KRAS_5−6/8−14_ G12V complex, the side chain of the P4 V residue is available to mediate hydrophobic contact for TCR recognition. Also, in both complexes there is a possibility of hydrogen bonding interactions between the main chain carbonyl and amide groups of P4 and P5 residues with the TCR (Figures 8A–C). Hence, our analysis of the crystal structure suggests that both the spliced KRAS_5−6/8−14_ G12V and the non-spliced KRAS_5−14_ G12V epitope candidates can be contacted by the same TCR at their P4 site, thereby promoting cross reactivity.

On the other hand, even though some potential TCR cross reactivity exists toward both spliced and non-spliced epitope candidates, the structural superposition of both peptide complexes revealed deviation in their peptide conformation at the region where the TCR interaction is expected to happen (Figure 8C). Hence, depending on the direction that the TCR encounters in the HLA-I-peptide complex, there might be a definite possibility of having TCRs that exhibit preference or exclusive binding toward either the non-spliced or the spliced epitope, rather than recognizing both of them. As the KRAS_5−6/8−14_ G12V possess three peptide residues, P4 V, P5 V, and P6 G, that can mediate both hydrogen bonding and van der Waals contacts with a TCR, whilst the spliced peptide contains only P4 V and P5 G residues; hence, we can speculate that TCRs more likely will have selectivity and specificity for one of the two epitope candidates.

## Discussion

Epitope discovery is an essential first step for antigen-targeted immunotherapies against cancer, infection and some autoimmune diseases. In the last decade, several studies proposed strategies to achieve this, especially in light of anti-cancer immunotherapies. The majority of these studies identify epitope candidates in HLA-I immunopeptidomes eluted from cells. Although this strategy guarantees that the identified epitope is presented at the cell surface, it cannot include all targetable epitopes because of its relative low sensitivity (51). TCRs can still be considered more sensitive than MS-based methods and can sense even a few epitope molecules bound to HLA-I molecules to trigger cytotoxic responses. There are several examples of epitopes that were not identified by analytical methods based on HLA-I immunopeptidomes of cells, but were well-recognized by specific CTLs. The pipeline that we proposed here tries to circumvent this problem. While starting from a large number of theoretical epitope candidates, the pipeline narrows them down to a few selected candidates step by step. One of the advantages of our strategy is that its sequential steps could be exchanged and adapted to the specific requirements of a given application. For instance, in this study we developed a model to rank antigens by their potential over-representation in HLA-I immunopeptidomes considering four protein features, without including any cell-specific assays, such as transcriptome or intracellular proteome analysis. If such data was available, our pipeline could use more complex algorithms, such as that published by Pearson et al. (37), and likely reach a more in-depth antigen selection.

The same principle of flexible structure and interchangeable steps could be applied to the “*in vitro* selection” section of our pipeline. In this study, we tested *in vitro* three steps of the HLA-I APP pathway: proteasome-mediated generation; TAP-mediated transport into the ER lumen; and the efficient binding to the selected HLA-I variant. While some epitopes may be presented by HLA-I molecules in a proteasome- and TAP-independent fashion, the majority of HLA-I-restricted epitopes depends on these two steps.

Efficient binding to the selected HLA-I molecule is, on the contrary, mandatory. However, although a threshold of 500 nM would capture ~85% of all HLA-I-bound peptides (52, 53), it is still an open question what the optimal IC_50_ threshold is to define a “good epitope target” for ATT. The most determining factor could be the off-rate of peptide binding, a feature that we likely determine indirectly via IC_50_, because poor and good peptides have been reported to have similar on-rates but different off-rates (54).

Of course, the “*in vitro* selection” section of our pipeline could enlist other APP steps such as tapasin-dependency, cytosolic peptidase and ERAP trimming, etc. (2, 55), which could be selectively chosen based on tumor features and the known APP pathway of the target antigen.

Our pipeline identifies epitope candidates which shall further be validated by isolating specific CTL clones and their TCRs and using them to confirm that the epitope candidates are produced *in cellulo* and eventually *in vivo*. There are several strategies to this end. For example, the KRAS_5−6/8−14_ spliced epitope candidate here identified has been validated in collaboration with Blankenstein et al. (56). Specific TCRs have been isolated from humanized AB*ab*DII mice (57), cloned into expressing vectors and transduced into human PBMCs. Transduced human CD8^+^ T cells selectively recognized the KRAS_5−6/8−14_ spliced epitope. They also recognized human cancer cell lines expressing KRAS G12V antigen and HLA-A^*^02:01 complex and release IFNγ. They do not recognize a cancer cell line expressing the wild type KRAS G12 protein and the HLA-A^*^02:01 complex. These outcomes validated the KRAS_5−6/8−14_ spliced epitope candidate as a genuine epitope (56). We tried to identify the KRAS_5−6/8−14_ G12V spliced and the KRAS_5−14_ G12V non-spliced epitope candidates through the MS measurement of the HLA-I immunopeptidomes of the SW480 pancreatic adenocarcinoma cell line, which expresses the HLA-A^*^02:01 complex and the KRAS G12V mutated protein (42). Despite CTL clones could recognize both epitope candidates presented by cancer cell lines (42, 56), none of the two peptides was identified in the SW480-derived HLA-I immunopeptidomes (data not shown), thereby confirming the usefulness of the pipeline described here.

Another advantage of our pipeline is its ability to select and identify proteasome-generated spliced epitope candidates, which we and other groups have found to represent a sizeable pool of immunologically relevant epitopes, especially within the framework of anti-cancer immunotherapies (3, 11, 12, 20, 21, 44, 58). The PCPS reaction was shown to generate a large number and a significant amount of spliced peptides in the *in vitro* processing of KRAS_2−35_ neoantigen by 20S proteasomes. This suggests that we might have previously underestimated PCPS frequency in the *in vitro* proteasome digestions, likely due to the low MS sensitivity available at that time (23).

The benefit of including these unconventional epitopes in our pipeline is evident. The recurrent KRAS G12V mutation can be efficiently presented by HLA-A^*^02:01 complexes only through spliced peptides. TCRs specific to this spliced epitope candidate could be used to treat around 15–20% of colorectal cancer and pancreatic adenocarcinoma by ATT. According to our analysis, the G12V mutation promotes not only the binding affinity of the KRAS_5−6/8−14_ G12V spliced epitope candidate to HLA-A^*^02:01 complex, but also the splicing reaction at that site, since we did not identify the KRAS_5−6/8−14_ G12 spliced peptide in the *in vitro* digestion of wild type KRAS_2−35_ G12 by 20S proteasomes. The KRAS_5−6/8−14_ G12V sequence [KL][VVGA**V**GV] cannot be generated by any other human protein by peptide hydrolysis or *cis* peptide splicing (data not shown), thereby defining it as a unique neoepitope. In the KRAS_2−35_ G12V polypeptide digestion, we also identified the non-spliced epitope candidate KRAS_5−14_ G12V, which was shown to be recognized by PBMCs of pancreatic adenocarcinoma patients (42). For a cancer-targeted strategy, it would be informative to perform *in vitro* digestions using proteasome isoforms recapitulating those present in the target cancer, since they vary from tumor to tumor with implications for the quantity (and perhaps the quality) of peptide produced (4, 5, 27). Both KRAS_5−6/8−14_ G12V and KRAS_5−14_ G12V epitope candidates are efficiently transported by TAP into the ER lumen, which is then no bottleneck. The non-spliced epitope candidate, however, binds HLA-A^*^02:01 less efficiently than the spliced epitope candidate with an IC_50_ larger than 300 nM, which could be higher than the binding affinity currently suggested for immunodominant epitopes. According to analysis of the HLA-A^*^02:01-peptide crystal structures, the two epitope candidates differ in the region exposed to TCRs. Therefore, we would expect that unique CD8^+^ T cell clones could recognize these, although cross-reactive TCRs cannot be excluded. In the case of cross-reactivity, the immunodominance of the spliced epitope over the non-spliced epitope might be favored by the higher binding affinity to HLA-A^*^02:01 complex. We do not have enough information about the other steps of their APP pathways, including production by cancer-associated proteasome isoforms, to conclude about presentation in cancer patients. Yet, our pipeline allows identification of potential new neoepitopes derived from peptide splicing that are unique for a driver in oncogenesis, KRAS G12V. Such epitopes could be critical in new vaccination approaches for the related tumors.

## Data Availability Statement

All datasets generated for this study are either included in the article/Supplementary Material or deposited in online repositories, as clarified in the Materials and Methods section.

## Author Contributions

MM and JL developed the whole project, performed and supervised the data analysis and data generation, and wrote the manuscript. DZ supervised the crystal structure analysis and wrote the cognate text. JS, JN, and AS supervised part of the analysis, wrote, and edited the text. AM, GY, AB, and RC performed and analyzed part of the experiments and proofread the manuscript. SH and DP carried out QPuB analysis and proofread the manuscript. HU supervised MS measurement and optimization.

### Conflict of Interest

MM and JL are co-inventors of the spliced epitope and specific TCRs protected by the patent PCT/EP2019/050027. The remaining authors declare that the research was conducted in the absence of any commercial or financial relationships that could be construed as a potential conflict of interest.
