# Supplementary material for: An in silico—in vitro Pipeline Identifying an HLA-A*02:01+ KRAS G12V+ Spliced Epitope Candidate for a Broad Tumor-Immune Response in Cancer Patients
Source: Front Immunol. 2019 Nov 15;10:2572. doi: 10.3389/fimmu.2019.02572 (PMC6872521; doi:10.3389/fimmu.2019.02572)
Supplement: Supplementary file 1 [file Data_Sheet_1.pdf]

An *in silico - in vitro* pipeline identifying an HLA-A*02:01+ KRAS G12V+ spliced epitope candidate for a broad tumor - immune response in cancer patients

Mishto *et al.*

Supplementary Table S1	MS inclusion list
Supplementary Table S2	Synthetic peptide list
Supplementary Table S3	Crystal structure data collection and refinement statistics
Supplementary Figure 1	MS/MS spectra of the non-spliced epitope candidate $KRAS_{5\text{-}14}$ G12V

Name (KRAS G12V)	sequence	m/z	charge
28_32_11_14	FVDEYAVGV	998.4829	+1
28_33_12_14	FVDEYDVGV	1042.4728	+1
28_34_11_12	FVDEYDPAV	1054.4728	+1
28_32_9_12	FVDEYVGAV	998.4829	+1
28_31_10_14	FVDEGAVGV	892.4411	+1
28_31_8_12	FVDEVVGAV	934.488	+1
24_29_12_14	IQNHFVVGV	1012.5574	+1
28_32_8_12	FVDEYVVGAV	1097.5514	+1
22_28_11_12	QLIQNHFAV	1069.5789	+1
18_24_12_14	ALTIQLIVGV	1026.6558	+1
28_31_7_12	FVDEVVVGAV	1033.5564	+1
28_30_9_14	FVDVGAVGV	862.4669	+1
5_9_11_14	KLVVVAVGV	883.5975	+1
24_28_11_14	IQNHFAVGV	984.5261	+1
5_6_8_14	KLVVGAVGV	841.5506	+1
5_6_6_12	KLLVVVGAV	897.6132	+1
7_12_27_29	VVVGAVHFV	344.6589	+1
18_23_12_14	ALTIQLVGV	913.5717	+1
28_33_10_12	FVDEYDGAV	1014.4415	+1
28_33_11_14	FVDEYDAVGV	1113.5099	+1
28_34_12_14	FVDEYDPVGV	1139.5255	+1
22_25_10_14	QLIQGAVGV	884.52	+1
28_32_10_14	FVDEYGAVGV	1055.5044	+1
18_24_11_12	ALTIQLIAV	941.603	+1
28_33_9_12	FVDEYDVGAV	1113.5099	+1
18_19_8_14	ALVVGAVGV	784.4927	+1
28_28_6_14	FLVVVGAVGV	959.5924	+1
22_29_11_12	QLIQNHFVAV	1168.6473	+1
28_28_7_14	FVVVGAVGV	846.5084	+1
18_25_11_12	ALTIQLIQAV	1069.6616	+1
28_35_11_12	FVDEYDPTAV	1155.5204	+1
28_31_9_14	FVDEVGAVGV	991.5095	+1
22_24_9_14	QLIVGAVGV	855.5298	+1
18_19_6_12	ALLVVVGAV	840.5553	+1
4_4_6_14	YLVVVGAVGV	975.5873	+1
23_29_11_12	LIQNHFVAV	1040.5887	+1
5_10_12_14	KLVVVGVGV	869.5819	+1
28_30_7_12	FVDVVVGAV	345.6652	+1
21_28_11_12	IQLIQNHFAV	1182.663	+1
18_23_11_14	ALTIQLAVGV	984.6088	+1
4_6_8_14	YKLVVGAVGV	1004.6139	+1
7_12_12_14	VVVGAVVGV	798.5084	+1
4_4_7_14	YVVVGAVGV	862.5033	+1
19_24_12_14	LTIQLIVGV	955.6186	+1
5_7_7_12	KLVVVVGAV	883.5975	+1
5_7_6_12	KLVLVVVGAV	996.6816	+1
21_25_10_14	IQLIQGAVGV	997.6041	+1
28_32_11_14	FVDEYAVGV	499.74545	+2
28_33_12_14	FVDEYDVGV	521.7404	+2
28_34_11_12	FVDEYDPAV	527.7404	+2
28_32_9_12	FVDEYVGAV	499.74545	+2
28_31_10_14	FVDEGAVGV	446.72455	+2
28_31_8_12	FVDEVVGAV	467.748	+2
24_29_12_14	IQNHFVVGV	506.7827	+2
28_32_8_12	FVDEYVVGAV	549.2797	+2
22_28_11_12	QLIQNHFAV	535.29345	+2
18_24_12_14	ALTIQLIVGV	513.8319	+2
28_31_7_12	FVDEVVVGAV	517.2822	+2
28_30_9_14	FVDVGAVGV	431.73745	+2
5_9_11_14	KLVVVAVGV	442.30275	+2
24_28_11_14	IQNHFAVGV	492.76705	+2
5_6_8_14	KLVVGAVGV	421.2793	+2
5_6_6_12	KLLVVVGAV	449.3106	+2
7_12_27_29	VVVGAVHFV	172.83345	+2
18_23_12_14	ALTIQLVGV	457.28985	+2
28_33_10_12	FVDEYDGAV	507.72475	+2
28_33_11_14	FVDEYDAVGV	557.25895	+2
28_34_12_14	FVDEYDPVGV	570.26675	+2
22_25_10_14	QLIQGAVGV	442.764	+2
28_32_10_14	FVDEYGAVGV	528.2562	+2
18_24_11_12	ALTIQLIAV	471.3055	+2

28_33_9_12	FVDEYDVGAV	557.25895	+2
18_19_8_14	ALVVGAVGV	392.75035	+2
28_28_6_14	FLVVVGAVGV	480.3002	+2
22_29_11_12	QLIQNHFVAV	584.82765	+2
28_28_7_14	FVVVGAVGV	423.7582	+2
18_25_11_12	ALTIQLIQAV	535.3348	+2
28_35_11_12	FVDEYDPTAV	578.2642	+2
28_31_9_14	FVDEVGAVGV	496.25875	+2
22_24_9_14	QLIVGAVGV	428.2689	+2
18_19_6_12	ALLVVVGAV	420.78165	+2
4_4_6_14	YLVVVGAVGV	488.29765	+2
23_29_11_12	LIQNHFVAV	520.79835	+2
5_10_12_14	KLVVVGVGV	435.29495	+2
28_30_7_12	FVDVVVGAV	173.3366	+2
21_28_11_12	IQLIQNHFAV	591.8355	+2
18_23_11_14	ALTIQLAVGV	492.8084	+2
4_6_8_14	YKLVVGAVGV	502.81095	+2
7_12_12_14	VVVGAVVGV	399.7582	+2
4_4_7_14	YVVVGAVGV	431.75565	+2
19_24_12_14	LTIQLIVGV	478.3133	+2
5_7_7_12	KLVVVVGAV	442.30275	+2
5_7_6_12	KLVLVVVGAV	498.8448	+2
21_25_10_14	IQLIQGAVGV	499.30605	+2
28_32_11_14	FVDEYAVGV	333.499633	+3
28_33_12_14	FVDEYDVGV	348.162933	+3
28_34_11_12	FVDEYDPAV	352.162933	+3
28_32_9_12	FVDEYVGAV	333.499633	+3
28_31_10_14	FVDEGAVGV	298.152367	+3
28_31_8_12	FVDEVVGAV	312.168	+3
24_29_12_14	IQNHFVVGV	338.191133	+3
28_32_8_12	FVDEYVVGAV	366.522467	+3
22_28_11_12	QLIQNHFAV	357.1983	+3
18_24_12_14	ALTIQLIVGV	342.8906	+3
28_31_7_12	FVDEVVVGAV	345.1908	+3
28_30_9_14	FVDVGAVGV	288.160967	+3
5_9_11_14	KLVVVAVGV	295.2045	+3
24_28_11_14	IQNHFAVGV	328.847367	+3
5_6_8_14	KLVVGAVGV	281.188867	+3
5_6_6_12	KLLVVVGAV	299.8764	+3
7_12_27_29	VVVGAVHFV	115.5583	+3
18_23_12_14	ALTIQLVGV	305.1959	+3
28_33_10_12	FVDEYDGAV	338.819167	+3
28_33_11_14	FVDEYDAVGV	371.841967	+3
28_34_12_14	FVDEYDPVGV	380.513833	+3
22_25_10_14	QLIQGAVGV	295.512	+3
28_32_10_14	FVDEYGAVGV	352.5068	+3
18_24_11_12	ALTIQLIAV	314.539667	+3
28_33_9_12	FVDEYDVGAV	371.841967	+3
18_19_8_14	ALVVGAVGV	262.169567	+3
28_28_6_14	FLVVVGAVGV	320.536133	+3
22_29_11_12	QLIQNHFVAV	390.2211	+3
28_28_7_14	FVVVGAVGV	282.841467	+3
18_25_11_12	ALTIQLIQAV	357.225867	+3
28_35_11_12	FVDEYDPTAV	385.845467	+3
28_31_9_14	FVDEVGAVGV	331.175167	+3
22_24_9_14	QLIVGAVGV	285.8486	+3
18_19_6_12	ALLVVVGAV	280.8571	+3
4_4_6_14	YLVVVGAVGV	325.867767	+3
23_29_11_12	LIQNHFVAV	347.5349	+3
5_10_12_14	KLVVVGVGV	290.532633	+3
28_30_7_12	FVDVVVGAV	115.893733	+3
21_28_11_12	IQLIQNHFAV	394.893	+3
18_23_11_14	ALTIQLAVGV	328.874933	+3
4_6_8_14	YKLVVGAVGV	335.5433	+3
7_12_12_14	VVVGAVVGV	266.841467	+3
4_4_7_14	YVVVGAVGV	288.1731	+3
19_24_12_14	LTIQLIVGV	319.211533	+3
5_7_7_12	KLVVVVGAV	295.2045	+3
5_7_6_12	KLVLVVVGAV	332.8992	+3
21_25_10_14	IQLIQGAVGV	333.2067	+3
28_32_11_14	FVDEYAVGV	250.376725	+4
28_33_12_14	FVDEYDVGV	261.3742	+4

28_34_11_12	FVDEYDPAV	264.3742	+4
28_32_9_12	FVDEYVGAV	250.376725	+4
28_31_10_14	FVDEGAVGV	223.866275	+4
28_31_8_12	FVDEVVGAV	234.378	+4
24_29_12_14	IQNHFVVGV	253.89535	+4
28_32_8_12	FVDEYVVGAV	275.14385	+4
22_28_11_12	QLIQNHFAV	268.150725	+4
18_24_12_14	ALTIQLIVGV	257.41995	+4
28_31_7_12	FVDEVVVGAV	259.1451	+4
28_30_9_14	FVDVGAVGV	216.372725	+4
5_9_11_14	KLVVVAVGV	221.655375	+4
24_28_11_14	IQNHFAVGV	246.887525	+4
5_6_8_14	KLVVGAVGV	211.14365	+4
5_6_6_12	KLLVVVGAV	225.1593	+4
7_12_27_29	VVVGAVHFV	86.920725	+4
18_23_12_14	ALTIQLVGV	229.148925	+4
28_33_10_12	FVDEYDGAV	254.366375	+4
28_33_11_14	FVDEYDAVGV	279.133475	+4
28_34_12_14	FVDEYDPVGV	285.637375	+4
22_25_10_14	QLIQGAVGV	221.886	+4
28_32_10_14	FVDEYGAVGV	264.6321	+4
18_24_11_12	ALTIQLIAV	236.15675	+4
28_33_9_12	FVDEYDVGAV	279.133475	+4
18_19_8_14	ALVVGAVGV	196.879175	+4
28_28_6_14	FLVVVGAVGV	240.6541	+4
22_29_11_12	QLIQNHFVAV	292.917825	+4
28_28_7_14	FVVVGAVGV	212.3831	+4
18_25_11_12	ALTIQLIQAV	268.1714	+4
28_35_11_12	FVDEYDPTAV	289.6361	+4
28_31_9_14	FVDEVGAVGV	248.633375	+4
22_24_9_14	QLIVGAVGV	214.63845	+4
18_19_6_12	ALLVVVGAV	210.894825	+4
4_4_6_14	YLVVVGAVGV	244.652825	+4
23_29_11_12	LIQNHFVAV	260.903175	+4
5_10_12_14	KLVVVGVGV	218.151475	+4
28_30_7_12	FVDVVVGAV	87.1723	+4
21_28_11_12	IQLIQNHFAV	296.42175	+4
18_23_11_14	ALTIQLAVGV	246.9082	+4
4_6_8_14	YKLVVGAVGV	251.909475	+4
7_12_12_14	VVVGAVVGV	200.3831	+4
4_4_7_14	YVVVGAVGV	216.381825	+4
19_24_12_14	LTIQLIVGV	239.66065	+4
5_7_7_12	KLVVVVGAV	221.655375	+4
5_7_6_12	KLVLVVVGAV	249.9264	+4
21_25_10_14	IQLIQGAVGV	250.157025	+4

Supplementary Table S1. MS inclusion list. List of m/z used for the targeted MS approach.

**Peptide**	**Type**	**Sequence**
$KRAS_{2-35}$ G12	substrate	TEYKLVVVGAGGVGKSALTIQLIQNHFVDEYDPT
$KRAS_{2-35}$ G12V	substrate	TEYKLVVVGA**V**GVGKSALTIQLIQNHFVDEYDPT
$KRAS_{5-6/8-14}$ G12V	spliced peptide	KLVVGA**V**GV
$KRAS_{5-14}$ G12V	non-spliced peptide	KLVVVGA**V**GV
#1		YLVVVGAVGV
#2		KLVVVAVGV
#3		FLHEDLEKI
#4		FLHEDTEKI
#5	TAP competing peptide	TVNKTERCY (fluorescin bound to Cys side chain)

Supplementary Table S2. Synthetic peptide list. List of synthetic peptides used in the study. The name of the peptide depicts the cognate antigen and its position.

**Data collection statistics**	HLA-A*02:01 – $KRAS_{5\text{-}6/8\text{-}14}$ G12V	HLA-A*02:01 – peptide #2	HLA-A*02:01 – peptide #1	HLA-A*02:01 – $KRAS_{5\text{-}14}$ G12V
PDB ID	6O4Y	6O4Z	6O51	6O53
Space group	$P2_1$	$P2_1$	$P2_1$	$P2_1$
Cell dimension				
a. b. c. (Å)	52.99. 80.86. 56.93	56.13. 79.69. 57.44	56.26. 79.46. 57.25	56.24. 79.63. 57.22
α. β. γ(°)	90.00. 113.32. 90.00	90.00. 116.27. 90.00	90.00. 115.96. 90.00	90.00. 116.12. 90.00
Resolution range (Å)<br>[outer shell]	40.00 – 1.58<br>(1.62 – 1.58)	40.00 – 1.50<br>(1.53 – 1.50)	40.00 – 1.55<br>(1.59 – 1.55)	50.00 – 1.40<br>(1.42 – 1.40)
No. of unique reflections	58659 (4114)	70589 (4571)	63926 (3897)	85391 (4213)
R_{meas} (%)	6.4 (42.9)	5.2 (58.9)	5.6 (45.7)	6.8 (53.0)
R_{pim} (%)	3.3 (24.2)	2.7 (32.2)	3.0 (26.5)	3.9 (32.3)
Multiplicity	3.4 (2.8)	3.6 (3.2)	3.3 (2.6)	2.8 (2.3)
Average I/σI	24.04 (1.7)	24.9 (1.73)	21.8(1.70)	28.7 (1.75)
Completeness (%)	96.5 (88.4)	97.8 (95.3)	97.0 (88.9)	96.5 (95.5)
**Refinement statistics**				
No. atoms	3480	3448	3623	3472
Protein	3095	3097	3076	3044
ligand	59	70	69	73
Water	304	252	437	331
Glycerol/Na/Cl/sulfate	22	29	41	31
Ramachandran plot (%)				
Favored	97.2	97.7	97.7	97.8
Allowed	2.53	2.28	1.97	2.17
Outliers	0.28	0	0.28	0
R.m.s. deviations				
Bonds (Å)	0.006	0.005	0.006	0.005
Angles (°)	1.299	1.260	1.283	1.241
B-factors (Å^2)				
Protein	19.9	18.4	18.4	18.4
ligand	17.9	18.3	27.1	25.7
Water	30.2	31.3	33.1	30.9
Glycerol/Na/Cl/sulfate	31.6	34.5	43.6	41.6
R factor (%)	17.9	17.4	19.2	18.8
R_{free} (%)	20.4	19.3	22.4	20.9

Supplementary Table S3. Crystal structure data collection and refinement statistics.

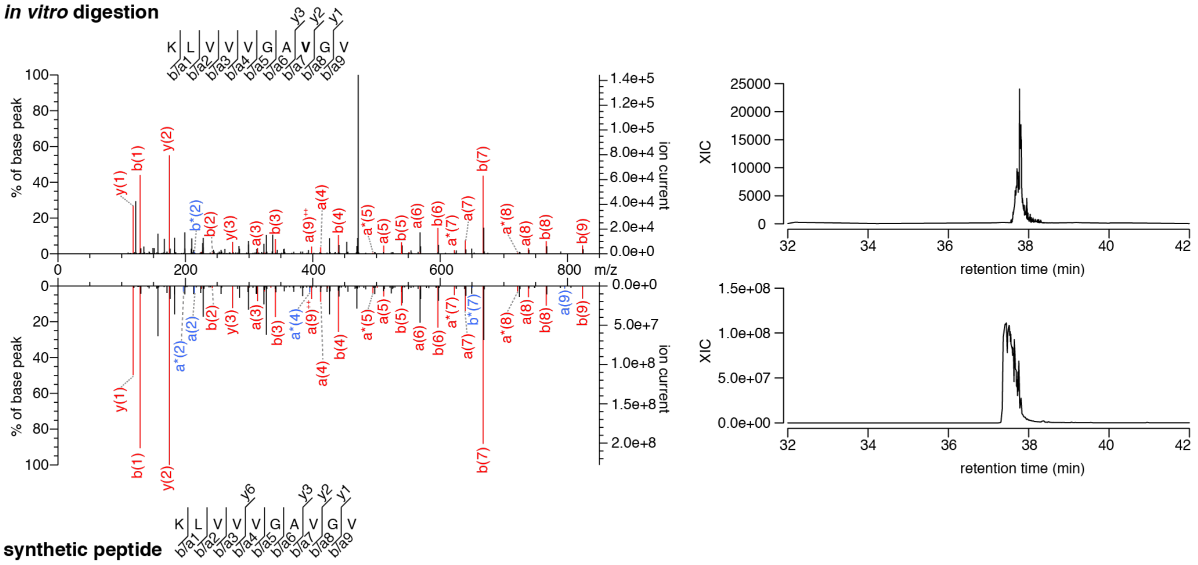

Supplementary Figure S1. MS/MS spectra of the KRAS$_{5-14}$ G12V non-spliced epitope candidate. MS/MS spectrum of the peptide KRAS$_{5-14}$ G12V [KLVVVGA**V**GV] identified in the 20 h *in vitro* digestion of the synthetic polypeptide KRAS$_{2-35}$ G12V with 20S proteasomes and the MS/MS spectrum of the cognate synthetic peptide (left). The peptide sequence is shown with the corresponding b-, a- and y-ions identified. The G12V mutation is depicted in bold. In the spectra, assigned peaks for b-, a- and y-ions are reported in color. Ion neutral loss of ammonia is symbolized by *. Red marked peaks are assigned in both the in vitro digestion detected MS/MS spectrum and the synthetic peptide MS/MS spectrum, while blue marked peaks are assigned only in one of the two spectra. The extracted ion chromatogram for the peptide identified in the *in vitro* digestion and the synthetic counterpart is plotted and indicates matching retention times for both peptides (right).
